# Periodontitis is associated with significant hepatic fibrosis in patients with non-alcoholic fatty liver disease

**DOI:** 10.1371/journal.pone.0185902

**Published:** 2017-12-08

**Authors:** William Alazawi, Eduardo Bernabe, David Tai, Tomasz Janicki, Polychronis Kemos, Salma Samsuddin, Wing-Kin Syn, David Gillam, Wendy Turner

**Affiliations:** 1 Blizard institute, Queen Mary, University of London, London, United Kingdom; 2 Institute of Dentistry, King’s College London, London, United Kingdom; 3 Dental Institute, Queen Mary, University of London, London, United Kingdom; 4 Section of Gastroenterology, Ralph H Johnson Veterans Affairs Medical Center, Charleston, SC, United States of America; 5 Division of Gastroenterology and Hepatology, Medical University of South Carolina, Charleston, SC, United States of America; Universita degli Studi di Verona, ITALY

## Abstract

**Background and aims:**

Non-alcoholic fatty liver disease (NAFLD) has a bidirectional association with metabolic syndrome. It affects up to 30% of the general population, 70% of individuals with diabetes and 90% with obesity. The main histological hallmark of progressive NAFLD is fibrosis. There is a bidirectional epidemiological link between periodontitis and metabolic syndrome. NAFLD, periodontitis and diabetes share common risk factors, are characterised by inflammation and associated with changes in commensal bacteria. Therefore we tested the hypothesis that periodontitis is associated with NAFLD and with significant fibrosis in two study groups.

**Methods:**

We analyzed data from a population-based survey and a patient-based study. NHANES III participants with abdominal ultrasound and sociodemographic, clinical, and oral examination data were extracted and appropriate weighting applied. In a separate patient-based study, consenting patients with biopsy-proved NAFLD (or with liver indices too mild to justify biopsy) underwent dental examination. Basic Periodontal Examination score was recorded.

**Results:**

In NHANES, periodontitis was significantly associated with steatosis in 8172 adults even after adjusting for sociodemographic factors. However, associations were fully explained after accounting for features of metabolic syndrome. In the patient-based study, periodontitis was significantly more common in patients with biopsy-proven NASH and any fibrosis (F0-F4) than without NASH (p = 0.009). Periodontitis was more common in patients with NASH and significant fibrosis (F2-4) than mild or no fibrosis (F0-1, p = 0.04).

**Conclusions:**

Complementary evidence from an epidemiological survey and a clinical study show that NAFLD is associated with periodontitis and that the association is stronger with significant liver fibrosis.

## Introduction

Non-alcoholic fatty liver disease (NAFLD) has a bidirectional association with the metabolic syndrome [1]. It affects up to 30% of the general population in the West, 70% of individuals with diabetes and up to 90% of those with obesity [2]. Diabetes is an independent risk factor for NAFLD [3] and the risk of NAFLD increases with rising fasting plasma glucose levels by between 10–20% per mmol/l increase[4, 5]. Patients at all stages of NAFLD are at increased risk of cardiovascular morbidity and mortality.

Periodontitis is a common public health problem worldwide [6–8]. Chronic inflammation in the supporting structures of the teeth is driven by a complex relationship between oral pathogenic bacteria, presence of the dental biofilm subgingivally and an exacerbated and dysregulated inflammatory response to this plaque biofilm. This can lead to the formation of pockets (abnormal increase in depth of the gingival sulcus), clinical attachment loss (loss of the periodontal support around a tooth), and eventually tooth loss. Periodontitis causes pain and discomfort in the mouth, has a significant impact on both overall- and oral health-related quality of life, and on health-associated economic costs [9].

Several epidemiological studies demonstrate a bidirectional association between periodontal disease and components of the metabolic syndrome [10], diabetes[11, 12], obesity[13, 14] and cardiovascular disease[15, 16]. Patients with diabetes have an increased risk of severe periodontitis (2.8-fold) and of radiographic bone loss (3.4-fold) [17–19]. Higher blood glucose levels are associated with more severe and progressive chronic periodontitis [20]. Reciprocally, oral pathogenic bacteria increase availability of fructose, and insulin resistance is worsened by the effect of periodontal inflammatory cytokines that are released systemically and impair insulin receptor signaling. Systemic inflammation and translocation of commensal bacteria are characteristic of periodontitis and are implicated in the progression of liver fibrosis in NAFLD [2].

Surprisingly, there have been no studies to date that assess the relationship between NAFLD and periodontal disease. NAFLD, periodontal disease and diabetes share risk factors such as age, diet and socioeconomic group [2, 21]. Therefore large patient cohorts are required in order to study independent association between the conditions, but very few datasets exist that combine both metabolic and oral health together with these potential covariables. Prospective studies of carefully phenotyped patients are particularly useful to study these associations in more detail and to begin to explore causality.

The National Health and Nutrition Examination Survey (NHANES) is a program of clinical studies that combines clinical and dental measurements with demographic, socioeconomic, dietary and health-related questions and with laboratory test results. The Third NHANES survey (1988 to 1994) used a stratified, clustered, probability sample design to obtain a representative sample of US households and including ultrasonography of the gallbladder in 14 797 adults aged 20–74 [22]. The Baltimore group undertook a comprehensive, retrospective review of these ultrasonographs to identify patients with NAFLD, enabling study of epidemiological associations of NAFLD.

In this study we have tested the hypothesis that there is an association of periodontitis with 1. hepatic steatosis in the NHANES III cohort and 2. with significant liver fibrosis in both NHANES III and in a prospective study of largely biopsy-proven NAFLD. We confirm the association with steatosis and demonstrate a gradient of periodontitis with worsening liver injury.

## Methods

### Population-based study

We used data from the third US National Health and Nutrition Examination Survey (NHANES III), conducted from 1988 to 1994 by the National Center for Health Statistics of the Centers for Disease Control and Prevention. NHANES III collected interview, examination and laboratory data from a representative sample of the civilian, non-institutionalized population. Gallbladder ultrasonography was included as part of the original NHANES III protocol for 14,797 participants aged 20–74 years and images were reassessed to grade hepatic steatosis as absent, mild, moderate or severe [3]. We excluded eligible participants who did not undergo a gallbladder ultrasound (n = 814) or whose ultrasound was ungradable (n = 127). NAFLD was defined as the presence of mild, moderate or severe steatosis in the absence of serum hepatitis B surface antigen or hepatitis C antibody positivity, serum transferrin saturation>50%, pregnancy and elevated alcohol consumption (>2 drinks/day for women or >3 drinks/day for men).

Current smokers were those who had smoked> = 100 cigarettes over their lifetime and smoked at the time of the interview; former smokers had smoked> = 100 cigarettes but did not currently smoke; and never smokers had not smoked > = 100 cigarettes in their lifetime. We defined diabetes based on self-reported physician diagnosis, medication use, fasting plasma glucose>126mg/dl or 2-hour oral glucose tolerance test>200mg/dL. Hypertension was defined based on self-reported physician diagnosis, medication use, systolic blood pressure> = 140mm Hg or diastolic blood pressure > = 90mm Hg. Hypercholesterolaemia was defined based on self-reported physician diagnosis, medication use and total cholesterol >240mg/dL.

Socio-demographic and medical data from interview questionnaires were gender, age, race/ethnicity, education level, poverty income ratio (PIR), smoking and medical history of diabetes, hypertension and hypercholesterolaemia. Where all parameters needed were available, we calculated the NAFLD fibrosis score (NFS) according to the formula: -1.675 + 0.037 x age (years) + 0.094 x BMI (kg/m^2^) + 1.13 x impaired fasting glycaemia or diabetes (yes = 1, no = 0) + 0.99 x AST/ALT ratio—0.013 x platelet (x10^9^/litre)– 0.66 x albumin (g/dl).

Examination data included body mass index and periodontal assessment. The following periodontal measures were calculated: extent (%) of sites with bleeding on probing (BoP), the extent of sites (%) with periodontal pocket depth (PPD) ≥5mm and the mean PPD across all sites, and the extent (%) of sites with clinical attachment loss (CAL) ≥3mm and the mean CAL across all sites. We used the following case definition of periodontitis: 2 sites with PPD ≥3mm from different sextants or 1 site with PPD≥5mm in any sextant. This definition was used to match as closely as possible and within the limits of NHANES III coding, to the definition chosen for the clinical study (see below). Dietary data included the total caloric intake (kcal), total saturated fatty acids (gm), caffeine (mg) and fructose (gm) derived from 24-hours dietary recall.

Sera from 8,153 NHANES III participants aged 40+ years with complete data for serum IgG antibodies against 19 oral bacterial species were used in secondary analysis. We created bacteria antibody clusters utilizing previously reported groupings [23]: orange-blue (*Eubacterium nodatum* and *Actinomyces naeslundii)* yellow-orange (*Streptococcus intermedius*, *Streptococcus oralis*, *Streptococcus mutans*, *Fusobacterium nucleatum*, *Parvimonas micra* and *Capnocytophaga ochracea)*, red-green (*Tannerella forsythia*, *Treponema denticola*, *Aggregatibacter actinomycetemcomitans mix*, *Eikenella corrodens*, *Selenomonas noxia*, *Veillonella parvula* and *Campylobacter rectus)*, orange-red (*Prevotella melaninogenica*, *Prevotella intermedia*, *Prevotella nigrescens* and *Prevotella gingivalis mix)*.

Cluster scores were created from the sum of the natural log transformed values of antibody levels for each species in the cluster to form continuous measures.

All analyses were weighted and accounted for NHANES III complex survey design. We compared the composition of participants with and without steatosis using the Chi-square test for categorical variables and the t-test for continuous variables. The association between each periodontal measure and steatosis (and for periodontal pathogen clusters and steatosis) was evaluated in crude and adjusted models using logistic regression as the outcome (presence of steatosis) was a dichotomous variable. Odds ratios were therefore reported. We present crude models and models adjusted for sociodemographic (sex, age, race/ethnicity, education, PIR) and behavioural factors (smoking and diet). Thereafter, we evaluate the individual contribution of each clinical measure (diabetes, hypertension, hypercholesterolemia, BMI) and all jointly.

### Patient-based study (London cohort)

This project was approved by the National Research Ethic Service (Reference 14/WA/1142). Patients attending a specialist NAFLD clinic were invited to give written informed consent to take part in a cross-sectional study. Patients were excluded if they were under 18 years, had type 1 diabetes, had an existing diagnosis of non-NAFLD chronic liver disease, had active malignancy, had significant chronic inflammatory disease, were taking immunosuppression (apart from inhaled or short courses of systemic steroids) or were unable to consent. Patients’ liver diagnoses were made in routine clinical practice, hence only patients in whom a liver biopsy was clinically indicated have histological staging of liver disease. However, non-invasive tests have very high negative predictive value and therefore are reliable at excluding significant disease [24]. Therefore, for downstream analysis, we combined patients whose non-invasive liver scores were too low to justify biopsy with those with biopsy-proven simple steatosis.

We recorded the medical history including smoking and alcohol consumption habits, drug history and demographics (race, gender, age). Anthropometric measurement including body weight, body height, waist circumference were recorded. All patients underwent an oral examination including Basic Periodontal Examination (BPE which is based on periodontal pocketing) and collection of a saliva sample [25]. Periodontitis was defined as a BPE code of 3 (probing depth 3.5–5.5mm) in 2 or more sextants or a code of 4 (probing depth >5.5mm) in any sextant. Clinicopathological data (including liver biopsy results where this was done) were extracted from the electronic patient record and all patients underwent transient elastography (Fibroscan). Liver biopsies reported by a single histopathologist (in routine clinical care) were summarised according to the National Institutes of Health NASH clinical research network (Kleiner) criteria [26].

Two-sample t-tests were used to investigate the differences in continuous variables between the groups of interest. Fisher’s exact test was used to investigate associations between categorical/binary variables. Spearman test was used as an additional exploratory method for correlations between ordinal and/or binary with other variables. Odds ratio and relative risk tests were calculated to identify the direction and size of an effect.

## Results

### Hepatic steatosis is associated with periodontal disease

Hepatic ultrasound data were available for 12,331 participants, of whom 4,159 (33.7%) were excluded because of missing data on relevant covariates. The final analytical sample thus included 8,172 participants aged 20 to 74 years. Thirty six percent of participants had hepatic steatosis; those with steatosis were significantly older, more likely to be male, less well educated, of Mexican ethnicity, and former or current smokers (Table 1). Steatosis was significantly associated with features of the metabolic syndrome: diabetes, hypertension, hypercholesterolaemia and BMI category. Steatosis was more common among participants of Mexican ethnicity (43.5%) versus other ethnic groups (33.8% White or 30.2% Black participants, p<0.001), and in diabetic versus non-diabetic patients (66.6% vs 33%, p<0.001). However, we did not find any differences in recorded dietary intake or poverty income ratio. Adults with steatosis were significantly more likely to have evidence of periodontal disease with a higher percentage of oral examination sites with BoP, PPD> = 4mm or CAL> = 3mm (p<0.01, Table 1). Adults with steatosis were also more likely to have periodontitis (12 vs 9.9%, p = 0.04, Table 1).

**Table 1:** Characteristics of respondents in NHANES III with steatosis on ultrasound and no steatosis (n = 8172).

Variable		No steatosis		Steatosis		p value
		n	%	n	%	
Sex	Male	2388	46.9	1408	52.6	0.003
	Female	2854	53.1	1522	47.5	
Age	20–24 years	839	15.0	282	8.3	<0.001
	25–34 years	1612	30.7	667	26.2	
	35–44 years	1238	26.3	749	28.9	
	45–54 years	594	13.2	473	16.8	
	55–64 years	500	8.4	415	11.5	
	65–74 years	459	6.4	344	8.3	
Education	<12 years	1529	16.0	1141	20.5	0.002
	12 years	1737	32.9	924	35.0	
	>12 years	1976	51.1	865	44.5	
Poverty income	≤100%	1130	11.2	737	11.0	0.115
ratio	101–200%	1322	18.9	766	19.2	
	201–300%	998	19.4	590	23.8	
	301–400%	735	19.1	337	16.7	
	>400%	1057	31.4	500	29.3	
Race/ethnicty	White	1920	76.2	981	73.9	0.002
	Black	1625	11.1	704	9.2	
	Mexican	1465	5.1	1128	7.3	
	Other	232	7.7	117	9.5	
Smoking status	Never smoker	2845	50.6	1561	49.9	0.002
	Former smoker	1032	21.7	729	27.1	
	Current smoker	1365	27.7	640	22.9	
Calories, mean (SD), kcal		2259.4	(852.4)	2321.0	(948.2)	0.167
Total sat. fatty acids, mean (SD) gm		30.0	(15.4)	31.3	(18.4)	0.156
Caffeine, mean (SD) mg		259.8	(299.1)	237.4	(280.1)	0.084
Fructose, mean (SD) gm		28.9	(21.0)	28.0	(23.3)	0.316
Diabetes	No	5017	97.2	2482	89.3	<0.001
	Yes	225	2.8	448	10.7	
Hypertension	No	4049	81.3	1853	65.6	<0.001
	Yes	1193	18.7	1077	34.4	
Cholesterol	No	4005	75.0	2056	67.9	<0.001
	Yes	1237	25.0	874	32.1	
BMI	<25	2449	53.7	763	28.9	<0.001
	25–29.9	1833	32.3	951	32.8	
	≥30	960	14.0	1216	38.3	
Bleeding on probing, mean (SD) %		7.7	(10.4)	9.3	(12.6)	0.003
% of sites/mouth PPD≥4mm, mean (SD)		2.1	(5.2)	2.5	(5.8)	0.015
Mean PPD (SD), mm		1.4	(0.4)	1.5	(0.4)	0.001
% of sites/mouth CAL≥3mm, mean (SD)		6.9	(12.4)	9.3	(15.6)	0.001
Mean CAL (SD), mm		0.9	(0.7)	1.0	(0.8)	0.004
Periodontitis	Non-case	4463	90.1	2419	88.0	0.041
	Case	779	9.9	511	12.0	

SD—standard deviation, BMI–body mass index, BoP–bleeding on probing, PPD–periodontal pocket depth, CAL–clinical attachment loss.

The crude odds ratio (OR) for steatosis was comparable and statistically significant for all 5 markers of periodontitis (Table 2). After adjusting for sociodemographic factors, only bleeding on probing and mean PPD remained significantly associated with steatosis. Significance was lost when adjusting for other components of the metabolic syndrome.

**Table 2:** Models for the association between indicators of periodontitis and steatosis (n = 8172).

Model	BoP (%)		PPD≥4mm (%)		Mean PPD		CAL≥3mm (%)		Mean CAL	
	OR	[95% CI]	OR	[95% CI]	OR	[95% CI]	OR	[95% CI]	OR	[95% CI]
Model 1	1.10	[1.04–1.17]**	1.06	[1.01–1.10]*	1.11	[1.05–1.18]**	1.13	[1.06–1.20]***	1.12	[1.04–1.21]**
Model 2	1.07	[1.00–1.14]*	1.03	[0.98–1.08]	1.08	[1.00–1.17]*	1.05	[0.97–1.14]	1.01	[0.92–1.11]
Model 3A	1.06	[0.99–1.13]	1.01	[0.96–1.07]	1.07	[0.99–1.16]	1.04	[0.95–1.13]	0.99	[0.90–1.09]
Model 3B	1.06	[0.99–1.12]	1.03	[0.98–1.08]	1.07	[0.99–1.17]	1.06	[0.97–1.15]	1.01	[0.91–1.12]
Model 3C	1.07	[1.00–1.14]	1.03	[0.98–1.08]	1.08	[1.00–1.17]*	1.05	[0.97–1.14]	1.01	[0.91–1.11]
Model 3D	1.04	[0.98–1.12]	1.00	[0.95–1.05]	1.03	[0.94–1.12]	1.05	[0.97–1.15]	1.00	[0.90–1.11]
Model 3E	1.03	[0.97–1.10]	0.99	[0.94–1.05]	1.02	[0.93–1.11]	1.05	[0.96–1.15]	0.98	[0.88–1.10]

OR–odds ratio, BoP–bleeding on probing, PPD–periodontal pocket depth, CAL–clinical attachment loss. Model 1—unadjusted, Model 2—adjusted for demographic (sex, age groups, ethnicity), socioeconomic (PIR, education) and behavioural factors (diet and smoking). Model 3A: Model 2 + diabetes, Model 3B: Model 2 + hypertension; Model 3C: Model 2 + cholesterol; Model 3D: Model 2 + BMI groups; Model 3E: Model 2 + all clinical factors.

*p<0.05

** p<0.01

***p<0.001.

### Hepatic steatosis was associated with serum antibodies of periodontal bacteria

Serum levels of antibodies to known oral pathogenic bacteria were available for 3236 participants (S1 Table). Hepatic steatosis was weakly associated with antibodies to pathogens of the red-green cluster after adjustment for demographic, socioeconomic, behavioural and metabolic factors. Antibodies to two specific bacteria: *S*. *noxia* and *S*. *oralis* were strongly and significantly associated with steatosis (OR 1.13 and 1.14 respectively) even after adjustment for elements of the metabolic syndrome.

### Periodontal disease is more severe in patients with more advanced NAFLD

In the 2930 participants with steatosis in NHANES III, we calculated the NAFLD fibrosis score (NFS). Sufficient data were available to calculate the NFS in 2835 individuals. Participants with indeterminate or high-risk scores (NFS <-1.455) had significantly more CAL (greater proportion of sites with CAL> = 3mm and mean CAL) than those with low-risk NFS. However, no differences were found in terms BoP, extent of sites with PPD> = 4mm and mean PPD (Table 3).

**Table 3:** Characteristics of respondents in NHANES III with steatosis on ultrasound and low risk versus indeterminate / high risk scores by NAFLD fibrosis score (NFS, n = 2835).

Variable		NAFLD = Low		NAFLD = Inter/High		p value
		n	%	n	%	
Sex	Male	960	53.4	404	52.1	0.781
	Female	1057	46.6	414	47.9	
Age	20–24 years	267	10.4	7	0.4	<0.001
	25–34 years	602	32.0	53	9.2	
	35–44 years	593	31.6	128	21.2	
	45–54 years	301	15.7	151	19.7	
	55–64 years	176	7.7	225	23.1	
	65–74 years	78	2.6	254	26.4	
Education	<12 years	735	19.7	373	23.0	0.182
	12 years	658	34.6	238	37.9	
	>12 years	624	45.7	207	39.1	
Poverty income	≤100%	524	11.6	194	8.8	0.236
ratio	101–200%	522	18.5	222	21.8	
	201–300%	407	24.7	165	22.4	
	301–400%	241	17.1	78	14.8	
	>400%	323	28.1	159	32.1	
Race/ethnicty	White	641	73.0	303	75.7	0.098
	Black	444	8.2	228	12.0	
	Mexican	840	8.2	264	5.1	
	Other	92	10.7	23	7.1	
Smoking status	Never smoker	1103	50.6	411	46.82	<0.001
	Former smoker	423	23.48	284	39.22	
	Current smoker	491	25.92	123	13.96	
Calories, mean (SD), kcal		2380.5	(906.3)	2180.3	(1057.5)	0.013
Total sat. fatty acids, mean (SD) gm		32.4	(17.9)	28.7	(19.3)	0.009
Caffeine, mean (SD) mg		229.1	(231.0)	261.5	(415.6)	0.456
Fructose, mean (SD) gm		28.0	(22.3)	28.1	(26.1)	0.956
Diabetes	No	1892	95.5	509	69.9	<0.001
	Yes	125	4.5	309	30.1	
Hypertension	No	1454	71.9	340	45.4	<0.001
	Yes	563	28.1	478	54.6	
Cholesterol	No	1473	69.5	513	61.9	0.016
	Yes	544	30.5	305	38.1	
BMI	<25	648	34.1	89	12.0	<0.001
	25–29.9	696	34.2	226	28.0	
	≥30	673	31.7	503	60.1	
Bleeding on probing, mean (SD) %		9.0	(12.0)	10.4	(14.7)	0.063
% of sites/mouth PPD≥4mm, mean (SD)		2.3	(5.4)	3.0	(6.7)	0.153
Mean PPD (SD), mm		1.5	(0.4)	1.5	(0.5)	0.103
% of sites/mouth CAL≥3mm, mean (SD)		7.5	(13.6)	14.7	(20.9)	<0.001
Mean CAL (SD), mm		0.9	(0.7)	1.3	(1.0)	<0.001
Periodontitis	Non-case	1678	88.5	667	86.8	0.577
	Case	339	11.5	151	13.2	

SD—standard deviation, BMI–body mass index, BoP–bleeding on probing, PPD–periodontal pocket depth, CAL–clinical attachment loss.

### Periodontal disease is more common in patients with liver biopsy-proven significant fibrosis

**Fig 1:**
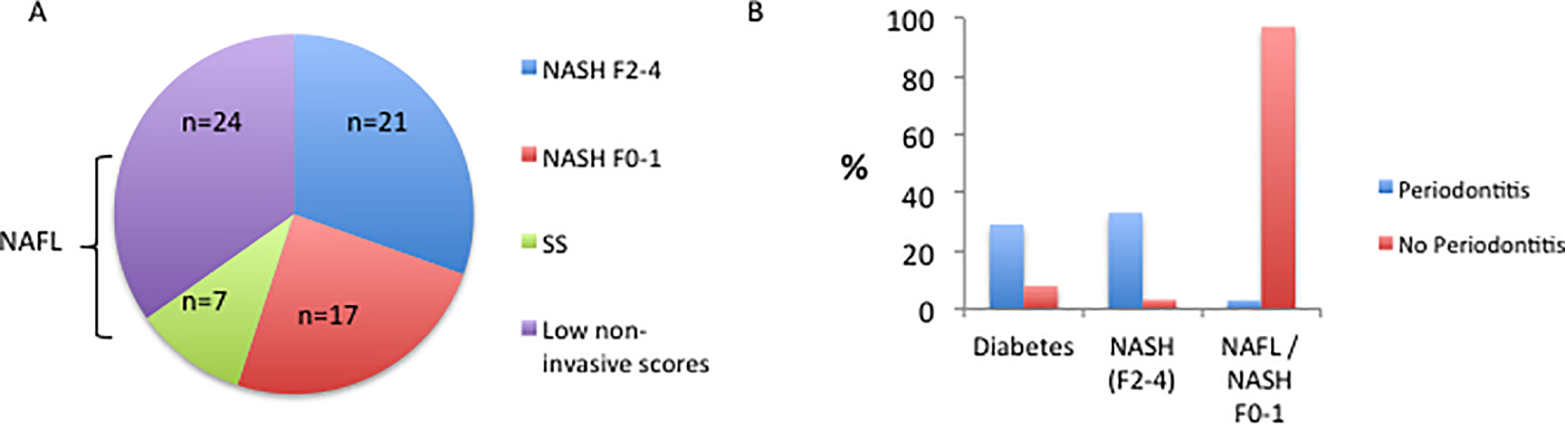
Prospective (London) cohort A. stage of liver disease and B. prevalence of periodontitis in patients with diabetes, NASH (F2-4) or NAFLD/NASH (F0-1).

The data from the NHANES cohort demonstrate an epidemiological association between oral health, hepatic steatosis and elements of the metabolic syndrome. The data also suggest that patients with more advanced liver disease have more severe indicators of periodontal disease. To examine this further, we conducted a prospective study of 69 patients with NAFLD in our centre. Of these, 45 (65%) had had a liver biopsy to stage the liver disease (Fig 1A). Of these, 21 had NASH with significant fibrosis (F2-4), 17 had NASH with minimal or no fibrosis (F0-1) and 7 had simple steatosis with no NASH or fibrosis. The remainder (n = 24, 35%) had clinical features that strongly indicated mild disease (low risk NAFLD fibrosis score and a low liver stiffness by valid Fibroscan reading) and in these people a liver biopsy was not clinically justified. These 24 patients were grouped with those who had a liver biopsy showing simple steatosis with no NASH or fibrosis (n = 7) and termed NAFL (n = 31).

Twelve patients (17.4%) had periodontitis. Patients with periodontitis were more likely to have diabetes than those without (75% vs 39%, p = 0.021, Table 4). Patients with periodontitis were more likely to have NASH of any stage (Table 4, 97% vs 42%, p = 0.0085) or NASH with fibrosis stages F2-4 (58% vs 25%, p = 0.03) than those without periodontitis.

**Table 4:** Characteristics of patients recruited in prospective (London) cohort with periodontitis or no periodontitis.

Characteristic	Periodontitis	No Periodontitis	p value
**Age**	49.2	50.6	p = ns
**Sex**	61.4	66.6	p = ns
**BMI**	31.5	30.7	p = ns
**Waist circumference (cm)**	105.6	101.3	p = ns
**Hip circumference (cm)**	115.7	104.0	0.035
**Ethnicity (% White)**	25	47.4	
**Diabetes (%)**	75	38.6	0.021
**Ever Smoked (%)**	25	21.4	p = ns
**Liver Stiffness (kPa)**	15.3	8.9	0.04
**NASH (F0-4, n)**	11	27	0.0085
**NAFL (n)**	1	30	

Reciprocally, periodontitis was significantly more common in patients with diabetes compared to those without (Fig 1B, 29% vs 8%, p = 0.03) and in patients with NASH with fibrosis stages F2-4, than in patients with NAFL (S2 Table, Fig 1B, 33% vs 3%, p = 0.005). Patients with NASH and no or mild fibrosis (F0-F1) had an intermediate prevalence of periodontitis (24%). There was no significant association of periodontitis with sex, ethnicity, blood pressure, or current smoking status.

In keeping with the tests of association above, we found that periodontitis correlated significantly with stage of liver disease (Table 5, NASH versus NAFL, p = 0.008) and the presence of diabetes (p = 0.01, Spearman correlations), but not with any of the other factors we recorded (age, ethnicity, sex, height, weight, BMI, waist and hip circumferences, blood pressure, liver stiffness or smoking).

**Table 5:** Spearman correlation of periodontitis with patient characteristics.

Characteristic	p value
**NASH vs NAFL**	0.008
**Age**	p = ns
**Sex**	p = ns
**Diabetes**	0.007
**Ethnicity**	0.24
**Smoking**	0.16

### Liver disease is independently associated with periodontitis

Having shown that NASH, diabetes and periodontitis are associated, we sought to determine the degree and direction of these associations. The odds ratio for having diabetes in patients with NASH compared to NAFL is 2.5 (95% CI 0.94–6.65). The odds ratio for having periodontitis in patients with NASH compared to NAFL is 12.2 (albeit with broad confidence interval; 95% CI: 1.48–101.0). The association remains significant even when controlling for diabetes: the relative risk of periodontitis in patients with NASH and diabetes is 1.54 (CI 1.04–2.28), and 1.14 (CI 0.95–1.38) in those without diabetes.

## Discussion

Epidemiological and clinical data reported here independently show a link between periodontitis and NAFLD, and this link is explained by features of metabolic syndrome including diabetes. Although we cannot demonstrate causality, both studies suggest a stronger association with periodontitis in patients with advanced, compared to mild, liver disease, fulfilling two of the Bradford Hill criteria (consistency and biological gradient) [27].

There is a growing literature reporting epidemiological association between oral and systemic health, focusing on diabetes, obesity and cardiovascular risk, however the potential link between periodontitis and NAFLD patients has received scant attention. Perceived barriers to such studies may include lack of infrastructure in most centres that can bring hepatological and dental assessment together in a research environment as we have done.

A major challenge in studying associations between oral and systemic health is that periodontitis can be clinically defined according to different measures of periodontal pathology [28, 29]. In general, diagnosis of periodontitis is based on clinical measurements such as probing depth, CAL and gingival inflammation. The BPE used in the patient-based study is a clinical tool that is specifically used to screen for periodontitis, where all teeth are examined with only the most severe score for pocket depth (but not CAL) in each sextant recorded. Therefore, it may under-estimate disease extent, severity and prevalence. The NHANES III study utilised a partial recording system for periodontal examination and is therefore likely to under-estimate prevalence of periodontitis [30]. Thus the parameters measured in the NHANES and the local cohorts are sufficiently different that detailed comparisons are difficult to make.

Determining steatosis by retrospective assessment of ultrasound in NHANES was a pivotal step in allowing researchers to study liver disease in this large survey [3]. However ultrasound (especially this retrospective analytical approach) is less sensitive in patients with <20% steatosis [31] and it does not provide an estimate of the degree of liver inflammation and fibrosis and we have gone some way to address this by applying the NAFLD fibrosis score. In keeping with previous studies, steatosis was more common among participants of Mexican ethnicity [32]. The peak age for steatosis in the NHANES cohort was relatively young (35–44 age group) and may reflect the overall age distribution of participants in this cohort. This in turn, may be related to our inclusion requirements that patients have liver ultrasound and dental examination. In the patient-based study, the majority of patients had a liver biopsy, widely accepted to be the gold standard in the assessment of NAFLD. Despite these differences, both studies show association between periodontitis, NAFLD and liver disease severity.

In the NHANES cohort, the statistical significance of the association between steatosis and oral health is lost after adjustment for metabolic factors. We hypothesise that the same mechanisms that underlie the association between oral health and diabetes are responsible, at least in part, for the association with NAFLD. Shared environmental risk factors (e.g. diet), the metabolic effect of oral bacteria increasing availability of certain sugars and insulin resistance may play a role. Hyperglycaemia and advanced glycation end-products are associated with increased destructive inflammatory cytokine production and impaired neutrophil clearance of bacteria. Inflammation or infection in the oral cavity has systemic effects that can affect insulin resistance and hepatic steatosis. Breaches in the epithelial barrier of the periodontal pocket can lead to low grade systemic bacteraemia and endotoxaemia [33]. These can activate inflammatory signaling pathways that de-phosphorylate insulin receptor substrate-1, reducing hepatocyte sensitivity to insulin. Similar mechanisms have been proposed to underlie the association of NAFLD with other inflammatory conditions such as psoriasis [34].

Although a recent randomised controlled trial of non-surgical periodontal intervention as a treatment for diabetes [35] was stopped early due to futility, many suggest that the inclusion criteria and inconsistencies in management of periodontitis in the study meant that the trial design may have played at least a part in this [36]. A Japanese pilot study has more recently shown that in patients with diabetes and chronic periodontitis, periodontal treatment improved glycated haemoglobin and, intriguingly, serum levels of gamma-glutamyl transpeptidase [37]. This raises the possibility of an interventional study to study these mechanisms in NAFLD.

Patients with decompensated liver disease awaiting transplantation frequently require oral assessment due to the high prevalence of periodontal infections in this group[38]. Oral bacteria are overrepresented in the fecal microbiome of patients with advanced liver disease [39]. Inoculation into the oral cavity of the major periodontal pathogen *P*. *gingivalis* in mice leads to alterations in the gut microbiome, gut tight junction integrity and insulin resistance [40]. PCR products of *P*. *gingivalis* (but not cultured bacteria) are more frequently detected in saliva from patients with NAFLD compared to controls [41]. Intravenous inoculation of P. gingivalis into mice fed a high fat diet resulted in increased weight gain and liver size due to steatosis compared to other oral bacteria. In the current study, we identify an association between steatosis and peripheral antibodies to a member of the Firmicutes phylum, *Selenomonas noxia* and *Streptococcus oralis*. There is relative over-abundance of Firmicutes in the gut microbiome of obese versus lean individuals [42] and the oral abundance of *S*. *noxia* in particular was strongly associated with obesity in a study of 313 obese women from Boston, MA [43].

Our data show an association between periodontitis and NAFLD in two independent studies. In addition, there is an association with significant liver fibrosis, even when controlling for diabetes in the prospective study. This has practical implications for the holistic care of patients with NAFLD. Taken in the context of other inflammatory associations with NAFLD, this novel observation generates testable mechanistic hypotheses regarding the progression of NAFLD and NASH with significant fibrosis.

## Supporting information

S1 Table Models for the association between periodontal pathogen clusters and steatosis (n = 3236). (DOCX)

S2 Table Characteristics of patients recruited in prospective (London) cohort. (DOCX)
