# Supplementary material for: Periodontitis is associated with significant hepatic fibrosis in patients with non-alcoholic fatty liver disease
Source: PLoS One. 2017 Dec 8;12(12):e0185902. doi: 10.1371/journal.pone.0185902 (PMC5722374; doi:10.1371/journal.pone.0185902)
Supplement: S1 Table — (DOCX) [file pone.0185902.s001.docx]

| **Model** | **Orange-red** | | **Red-green** | | **Yellow-orange** | | **Orange-blue** | |
| --- | --- | --- | --- | --- | --- | --- | --- | --- |
|  | **OR** | **[95% CI]** | **OR** | **[95% CI]** | **OR** | **[95% CI]** | **OR** | **[95% CI]** |
| Model 1 | 1.04 | [1.01-1.08]** | 1.03 | [1.01-1.05]** | 1.03 | [1.01-1.05]* | 1.01 | [0.94-1.08] |
| Model 2 | 1.03 | [1.00-1.07] | 1.03 | [1.01-1.05]** | 1.03 | [1.00-1.06]* | 1.02 | [0.95-1.09] |
| Model 3 | 1.02 | [0.98-1.07] | 1.03 | [1.00-1.05]* | 1.02 | [0.99-1.05] | 1.02 | [0.94-1.10] |
| Model 1 |  |  | 1.18 | [1.08-1.30]** |  |  |  |  |
| Model 2 |  |  | 1.18 | [1.06-1.30]** |  |  |  |  |
| Model 3 |  |  | 1.13 | [1.01-1.27]* |  |  |  |  |
| Model 1 |  |  |  |  | 1.16 | [1.05-1.28]** | |  |
| Model 2 |  |  |  |  | 1.17 | [1.04-1.31]** | |  |
| Model 3 |  |  |  |  | 1.14 | [1.01-1.29]* |  |  |

**S1 Table. Models for the association between periodontal pathogen clusters and steatosis (n=3236).** OR – odds ratio. CI – confidence interval. Model 1 - unadjusted, Model 2 - adjusted for demographic (sex, age groups, ethnicity), socioeconomic (PIR, education) and behavioural factors (diet and smoking).
