# Supplementary material for: Periodontitis is associated with significant hepatic fibrosis in patients with non-alcoholic fatty liver disease
Source: PLoS One. 2017 Dec 8;12(12):e0185902. doi: 10.1371/journal.pone.0185902 (PMC5722374; doi:10.1371/journal.pone.0185902)
Supplement: S2 Table — (DOCX) [file pone.0185902.s002.docx]

| Characteristic | F2-F4 | F0-F1 | p value |
| --- | --- | --- | --- |
| Age | 55.8 | 48.0 | 0.03 |
| Sex | 76.2 | 56.3 | p=ns |
| Weight in kg | 85.1 | 85.3 | p=ns |
| Height in cm | 166.0 | 166.6 | p=ns |
| BMI | 30.9 | 30.8 | p=ns |
| Waist circumference (cm) | 103.2 | 101.6 | p=ns |
| Hip circumference (cm) | 108.5 | 105.0 | p=ns |
| Blood pressure - systolic | 137.7 | 136.5 | p=ns |
| Ethnicity (% White) | 47.6 | 41.6 | p=ns |
| Diabetes (%) | 71.4 | 33.3 | 0.017 |
| Ever Smoked (%) | 29.4 | 20.8 | p=ns |
| Liver Stiffness (kPa) | 15.5 | 6.5 | 2.1x10^-5^ |
| Perio by BPE (>2 sextants with 3 or 1 with 4) | 33.3 | 10.4 | 0.035 |

**S2 Table. Characteristics of patients recruited in prospective (London) cohort** with NASH with significant fibrosis (F2-4) and patients with F0-F1 fibrosis (including those with NAFL and no NASH)
